# A rare case of Lung adenocarcinoma complicated with leptomeningeal and brain parenchymal metastasis: case report and review of the literature

**DOI:** 10.3389/fonc.2026.1884046

**Published:** 2026-07-29

**Authors:** Xiaobin Xu, Qianzheng Su, Zhaojin Cheng, Yin Li

**Affiliations:** Department of Neurosurgery, The Third Affiliated Hospital of Kunming Medical University, Yunnan Cancer Hospital, Peking University Cancer Hospital Yunnan, Kunming, Yunnan, China

**Keywords:** case report, intrathecal chemotherapy, leptomeningeal metastasis, NSCLC, targeted therapy

## Abstract

Brain metastasis (BM) represents a severe and common complication of advanced lung cancer, which is clinically categorized into brain parenchymal metastasis (BPM) and leptomeningeal metastasis (LM), with LM being the predominant subtype of meningeal involvement. In clinical practice, the concurrent occurrence of both parenchymal brain and leptomeningeal metastases in a single patient remains rare. Here, we report a case of an epidermal growth factor receptor (EGFR)-mutant NSCLC patient with simultaneous BPM and LM, along with a review of current research progress on the treatment of BM. The patient had previously undergone surgery and targeted therapy, and subsequently developed bone metastases, LM and BPM. After receiving ventriculoperitoneal shunting, intrathecal chemotherapy via an Ommaya reservoir, and resection of the brain metastatic lesion, the patient’s condition was effectively controlled. This case suggests that some EGFR-mutant NSCLC patients may still experience CNS progression during EGFR-tyrosine kinase inhibitor (TKI) therapy. Moreover, local therapy (surgery) combined with intrathecal chemotherapy may improve quality of life and prolong survival, thus representing a feasible treatment option.

## 1 Introduction

In patients with non-small cell lung cancer (NSCLC), BM occurs in approximately 23% to 36% of cases, and the majority of these patients present with isolated BPM (1). LM represents 11–20% of central nervous system (CNS) metastases, with the highest rates reported in melanoma and lung cancer (2). In EGFR-mutant NSCLC, the propensity for CNS dissemination is particularly pronounced, with cumulative incidence rates of BM reaching 40–60% over the disease course (3).However, the simultaneous occurrence of both BPM and LM in the same patient—while not exceptionally rare—remains infrequently documented in the literature.

LM involves tumor cells crossing the BBB, followed by diffuse dissemination through the CSF circulation or direct leptomeningeal infiltration (4), Unlike BPM, LM cells can both adhere and grow in suspension, enabling solid nodule formation and widespread CNS spread. In contrast, BPM relies on synergistic intrinsic tumor traits and extrinsic microenvironmental factors, driving rapid local lesion growth (5).

NSCLC is among the most prevalent solid tumors complicated by BM and LM. Patients present with diverse and non-specific neurological symptoms, mainly divided into three categories: intracranial hypertension (dizziness, headache, vomiting and papilledema), meningeal irritation (nuchal rigidity, Kernig sign and Brudzinski sign), and cranial nerve impairment. LM indicates an extremely poor prognosis and often leads to rapid mortality. Despite advances in modern therapeutic regimens that have extended overall survival (OS) from 1–3 months to 3–11 months (6), the clinical management of this devastating complication remains a formidable challenge.

We present a patient with EGFR-mutant NSCLC who developed sequential CNS progression. This case demonstrates a multimodal approach combining targeted therapy, ventriculoperitoneal shunting, Ommaya reservoir-based intrathecal chemotherapy, and surgical resection. By documenting this trajectory, we aim to provide insights into the feasibility of this combined strategy and review current literature on CNS metastasis management in EGFR-mutant NSCLC.

## 2 Case description

### 2.1 Initial diagnosis and surgical treatment (September 2023)

In September 2023, a 53-year-old male patient was admitted to the Department of Thoracic Surgery, Yunnan Cancer Hospital, because a routine physical examination had revealed a right lung mass. His medical history was unremarkable for hypertension, diabetes, or coronary heart disease. He had a 10-year smoking history. His performance status before admission was good, with a Karnofsky Performance Status score of 90. No significant abnormalities were found on admission physical examination, and the patient’s general condition was good.

**Figure 1:**
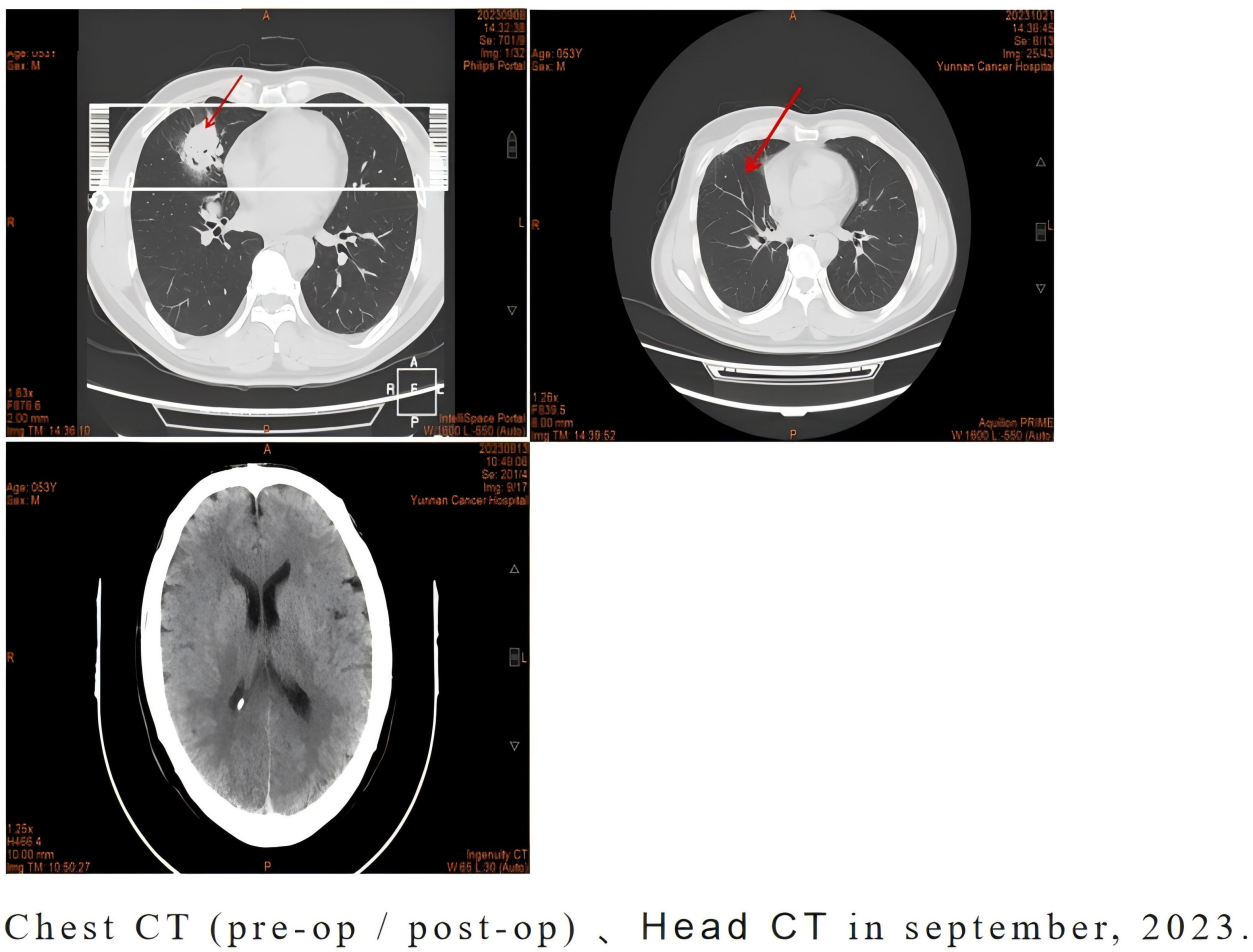
Chest CT (pre-op)/post-op), Head CT in September, 2023.

Chest spiral computed tomography (CT) performed on September 11, 2023, revealed a lobulated mass in the right middle lobe suggestive of malignancy, along with multiple nodules in both lungs and mediastinal lymphadenopathy, and Cranial CT on September 13, 2023, demonstrated no gross abnormalities (**Figure 1**). A needle biopsy was carried out on September 14, 2023; pathological examination demonstrated adenocarcinoma (**Supplementary Figure 1**), confirming the diagnosis of right middle lobe adenocarcinoma.

The patient had no contraindications to surgery, and his cardiopulmonary function was considered adequate to tolerate the procedure. On September 20, 2023, he underwent complete resection of the right middle lobe cancer via video-assisted thoracoscopic surgery (VATS) converted to thoracotomy. Postoperative pathological examination (September 24, 2023) diagnosed invasive adenocarcinoma of the right middle lobe (**Supplementary Figure 2**), with a pathological stage of pT2N2M0, IIIA.

The patient recovered well after surgery, with good wound healing. Follow-up chest CT showed no abnormalities (**Figure 1**), and he was discharged uneventfully on September 26, 2023.

### 2.2 Postoperative adjuvant targeted therapy and first progression (October 2023 – February 2024)

**Figure 2:**
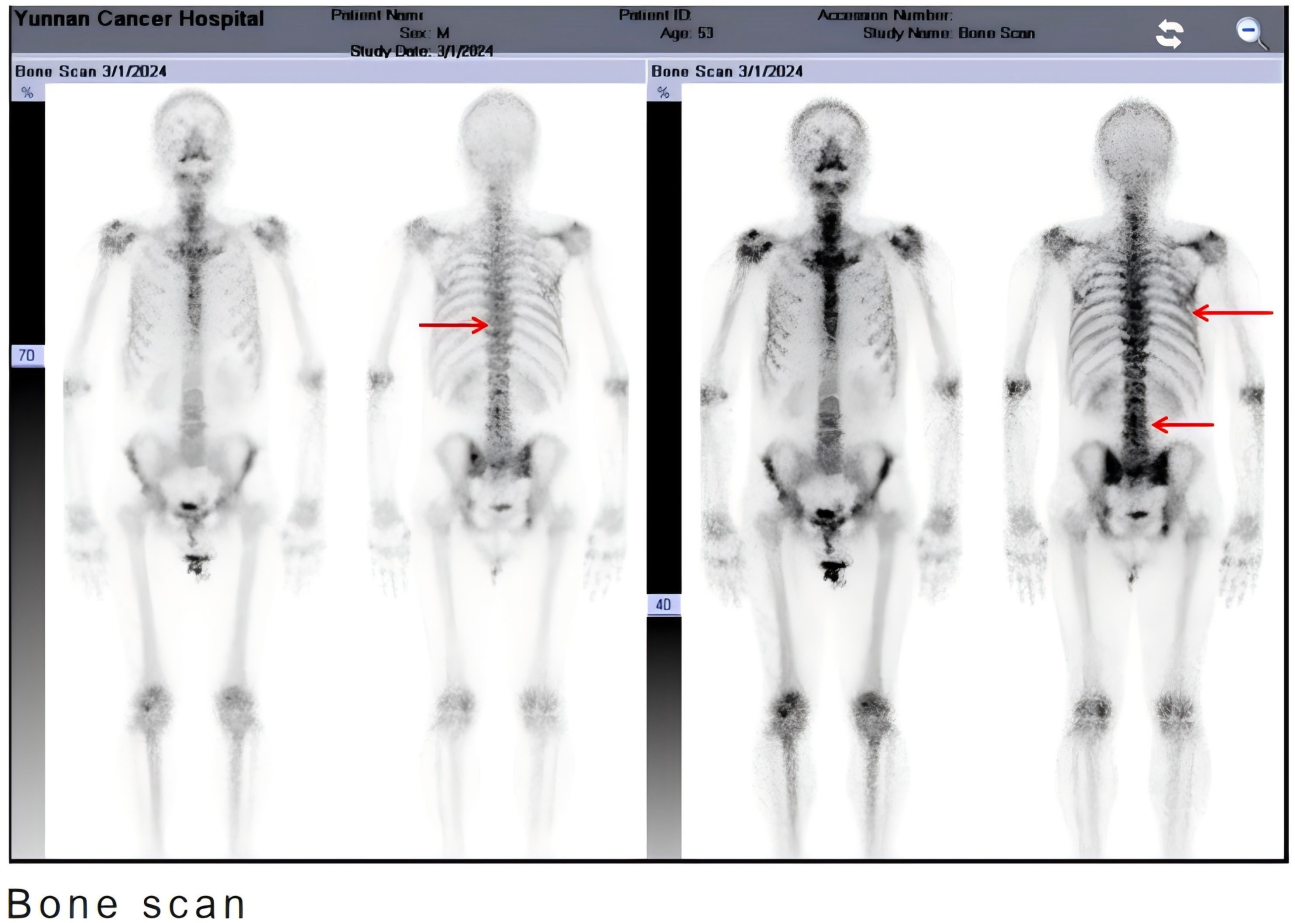
Bone scan.

On October 13, 2023, genetic testing revealed mutations in EGFR exon 21 p.Leu858Arg and TP53 exon 4 p.Ser90Profs, with variant allele frequencies of 31.32% and 19.98%, respectively (**Supplementary Figures 3, 4**). The patient was subsequently started on first-generation EGFR-TKI icotinib (125 mg orally three times daily) as targeted therapy, with regular follow-up. In February 2024, whole-body bone scintigraphy (**Figure 2**) suggested bone metastasis. Icotinib was therefore discontinued and replaced with the third-generation EGFR-TKI osimertinib (80 mg orally once daily). Thereafter, the patient’s condition remained relatively stable.

### 2.3 First neurosurgical admission and treatment (December 2024 – January 2025)

In December 2024, the patient developed dizziness and headache (worsened by activity) without an obvious trigger, accompanied by nausea, vomiting, limb weakness, and unsteady gait. During this period, he experienced one episode of syncope and was immediately taken to the emergency department of the First Affiliated Hospital of Kunming Medical University, where he received intravenous mannitol (250 mL of 20% solution, q8h) for osmotic diuresis, along with antiemetic and symptomatic supportive care. His symptoms transiently improved after this acute management, prompting further diagnostic workup. Subsequent investigations revealed no definite neoplastic lesions on brain magnetic resonance imaging (MRI) (**Supplementary Figure 5**); however, lumbar puncture and CSF cytology showed an intracranial pressure (ICP) of 300 mmH_2_O(normal supine reference range in adults: 70–200 mm H2O, equivalent to 5–15 mmHg) and atypical cells in the CSF, suggestive of LM (**Supplementary Figure 6**).

**Figure 3:**
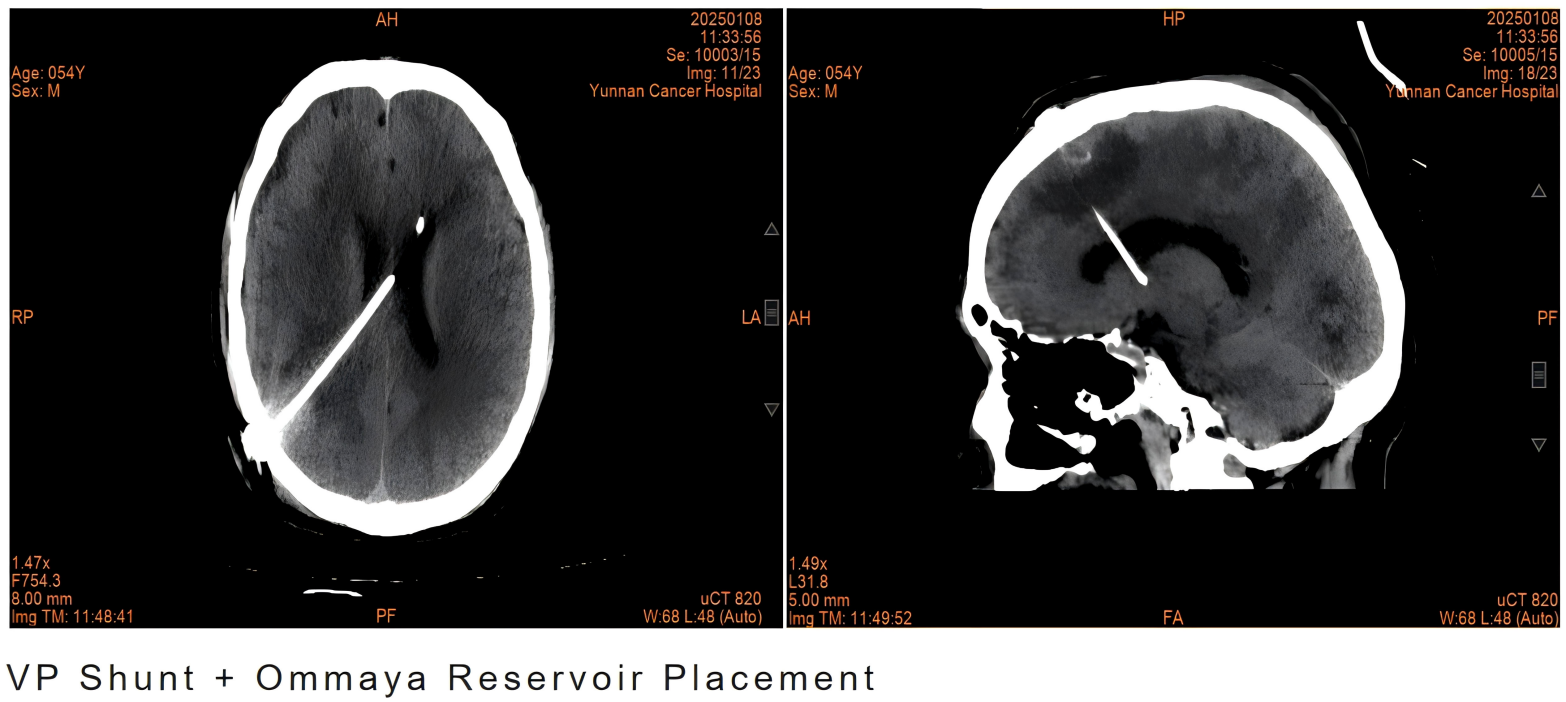
VP shunt + ommaya reservoir placement.

The patient was transferred to the Department of Neurosurgery of our hospital for further management. On admission, physical examination revealed an Eastern Cooperative Oncology Group performance status score of 1 and overt signs of elevated ICP. During the course of the disease, the patient experienced transient anisocoria and severe headache. To manage symptoms associated with diffuse intracranial hypertension, emergency ventriculoperitoneal shunting (VPS) combined with Ommaya reservoir placement was performed (to facilitate subsequent sequential intrathecal chemotherapy). Postoperative CT confirmed that the surgical outcome met expectations (**Figure 3**). On January 2, 2025, lumbar puncture showed that the ICP had decreased to 200 mmH_2_O. Thereafter, the patient’s clinical symptoms were markedly alleviated, and he was discharged uneventfully on January 12, 2025.

### 2.4 Long-term maintenance therapy: intrathecal chemotherapy (From February 2025)

The patient received intrathecal chemotherapy with pemetrexed disodium at a dose of 30 mg on days 1 and 8, with each cycle lasting 3 weeks. A total of 4 cycles were completed. Thereafter, maintenance therapy with osimertinib (80 mg orally once daily) was continued. The patient’s condition remained stable throughout the treatment period.

### 2.5 Third neurosurgical admission: resection of brain metastasis (September – October 2025)

**Figure 4:**
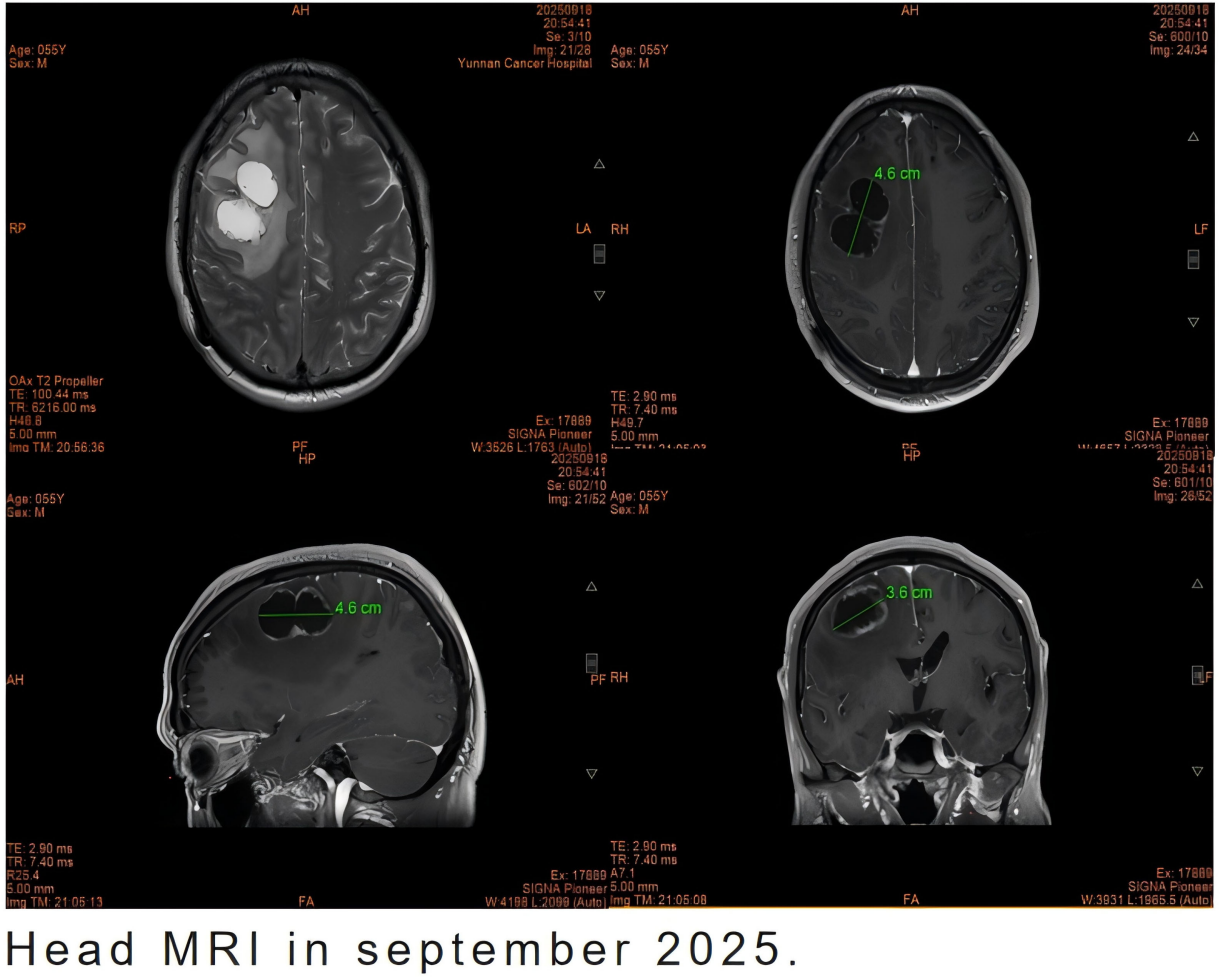
Head MRI in September 2025.

In September 2025, the patient experienced recurrent headache accompanied by one episode of seizure. Cranial MRI revealed a right frontal lobe space-occupying lesion measuring approximately 4.6 × 3.6 cm. T2-weighted/FLAIR sequences demonstrated hyperintense perilesional edema, and contrast-enhanced T1-weighted imaging showed heterogeneous enhancement of the lesion, suspicious for metastasis and midline shift, indicating focal intracranial hypertension (**Figure 4**).

**Figure 5:**
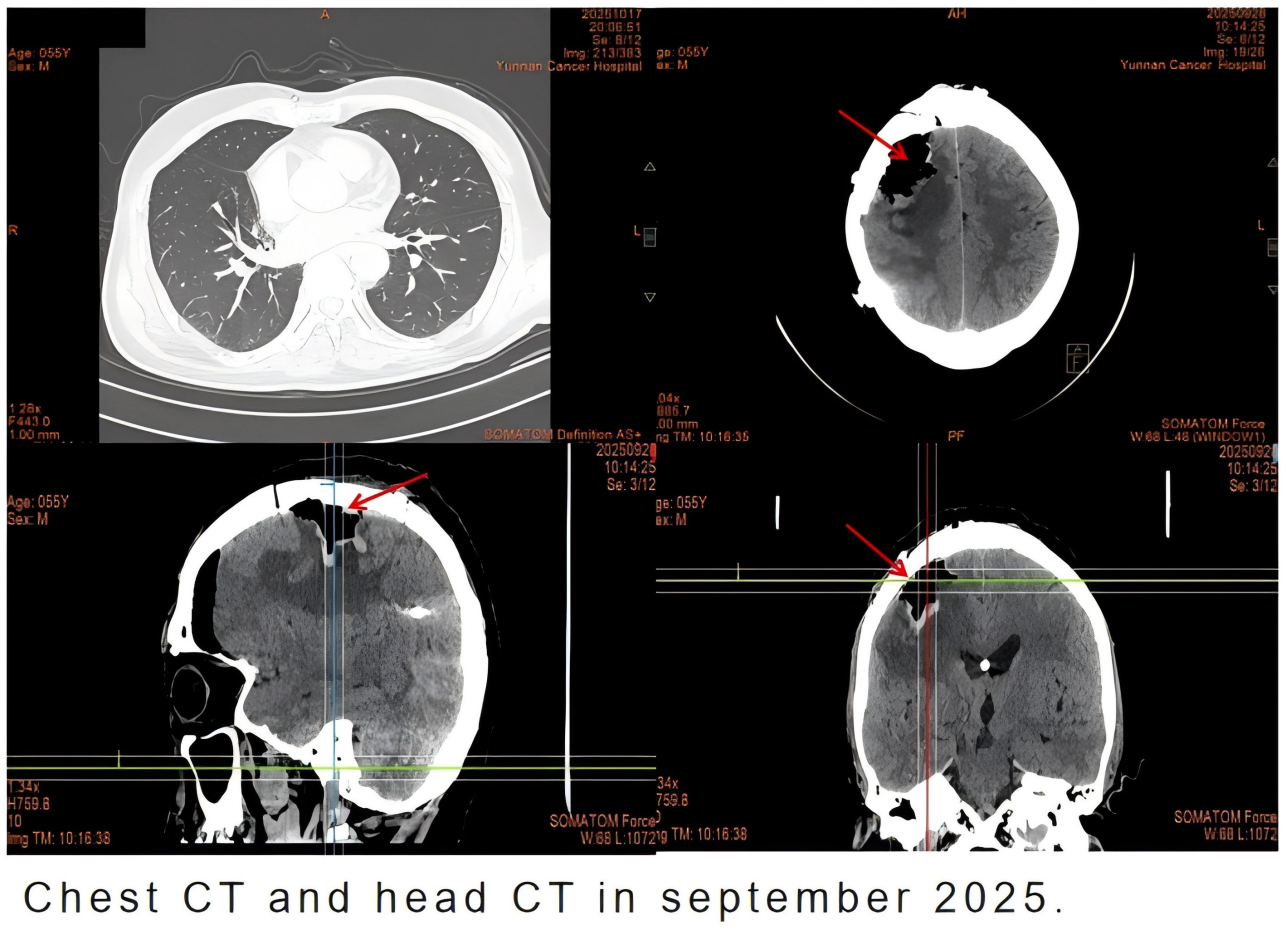
Chest CT and head CT in September 2025.

After excluding contraindications to surgery, the patient underwent resection of the right frontal lobe lesion under general anesthesia on September 27, 2025. Postoperative CT confirmed that the surgical outcome met expectations (**Figure 5**). Postoperative pathological examination (**Supplementary Figure 7**) confirmed metastatic adenocarcinoma. The patient recovered well after surgery and was uneventfully discharged on October 9, 2025.

### 2.6 Subsequent maintenance therapy

Subsequently, the patient continued to receive maintenance intrathecal pemetrexed (30 mg once every two weeks) via the Ommaya reservoir for a total of four cycles, concurrently with oral osimertinib (80 mg once daily) as systemic therapy.

### 2.7 Outcome and follow-up

Unfortunately, the patient passed away at his home in March 2026. The precise circumstances and immediate cause of death could not be determined, as no autopsy was performed and the patient did not seek emergency care during his final deterioration. Nevertheless, given the known history of progressive leptomeningeal and parenchymal metastases, the death was most likely attributable to progressive central nervous system involvement or consequent systemic decompensation. The overall survival from the initial diagnosis of stage IIIA NSCLC (September 2023) was 30 months, and the post-CNS-metastasis survival from the diagnosis of LM (December 2024) was 15 months.

## 3 Discussion

Leptomeningeal metastasis (LM) involves diffuse impairment of the entire neuraxis, from the cerebrum to the spinal cord. Approximately two-thirds of patients exhibit involvement of two or more anatomical regions (cerebrum, posterior fossa, and spinal cord) (7, 8). Common clinical manifestations include headache, projectile vomiting, diplopia, lower extremity weakness, and urinary retention (9). For malignant intracranial hypertension secondary to LM, ventriculoperitoneal shunting (VPS) is the first-line surgical intervention, which can effectively improve both clinical symptoms and quality of life (10).

It is important to emphasize that a negative cranial MRI does not exclude LM when clinical suspicion is high. MRI false-negative rates of 20%–30% have been reported in cytologically confirmed LM, and MRI may appear entirely normal even in patients with positive CSF cytology (11). Therefore, when symptoms suggest LM, a negative MRI should prompt CSF cytological evaluation rather than deferring further investigation. Our patient exemplifies this scenario, as LM was confirmed by CSF atypia despite an unremarkable initial cranial MRI, underscoring the complementary role of CSF analysis in cases with discordant clinical-radiological findings.

Intrathecal chemotherapy is an important palliative treatment modality. Implantation of an Ommaya reservoir allows direct intraventricular drug delivery, facilitating more homogeneous drug distribution throughout the cerebrospinal fluid space, with superior efficacy and better tolerability compared with repeated lumbar punctures (12, 13). Despite its advantages, the Ommaya reservoir carries a non-negligible risk of infectious complications, which remains a major concern in clinical practice. A 10-year retrospective study of adult patients with leptomeningeal metastases from solid tumors reported an Ommaya reservoir-related bacterial meningitis rate of 13.2% (5.9 infections per 10,000 Ommaya-days) (14).

Importantly, the risk of infection is closely associated with the frequency of reservoir access; one study found that infections occurred after a median of 6 (range 1–14) reservoir punctures (15).

In our patient, the Ommaya reservoir was accessed repeatedly for biweekly intrathecal pemetrexed administration over several months; although no infectious complication was documented during his treatment course, this experience underscores the importance of rigorous infection prevention measures and close monitoring for early signs of reservoir-related meningitis in patients receiving long-term intraventricular therapy.

The recommended dose of intrathecal pemetrexed is 10–50 mg per administration, given once or twice weekly (16, 17). Corticosteroids play a limited role in LM. The EANO-ESMO guidelines state that their efficacy in LM has not been specifically studied; thus, their use should be reserved for selected situations such as spinal cord compression or chemical meningitis, at the lowest effective dose (e.g., dexamethasone 4–8 mg/day) for the shortest possible duration (13).

Brain parenchymal metastasis (BPM) is the most common type of central nervous system (CNS) metastasis in NSCLC. For symptomatic BPM, local treatment modalities—including surgical resection, stereotactic radiosurgery (SRS), and/or radiotherapy—should be offered regardless of systemic therapy (18). Surgical resection is indicated not only for solitary lesions with significant mass effect, but also for oligometastatic or isolated lesions refractory to radiotherapy and systemic therapy (11). Postoperative SRS combined with surgery is a Grade I recommendation, as it reduces the risk of local recurrence compared with surgery alone (13).

### 3.1 Case summary

This EGFR/TP53-mutant NSCLC patient progressed from stage IIIA to IV, with early resistance to adjuvant icotinib and transient response to osimertinib. Multimodal therapy—VPS, intrathecal pemetrexed, and surgery—achieved 15-month post-LM and 30-month OS. This case underscores that CNS progression can occur on third-generation TKIs and that multidisciplinary intervention with local and intrathecal therapies may prolong survival. Early recognition and individualized treatment remain paramount.

### 3.2 Future perspectives

More innovative therapeutic strategies warrant further clinical exploration, such as osimertinib combined with chemotherapy(In the phase III TOP trial (19), osimertinib plus platinum-based chemotherapy achieved a median progression-free survival (PFS) of 34.0 months versus 15.6 months with osimertinib alone in patients with EGFR-mutant advanced NSCLC harboring concurrent TP53 mutations), amivantamab plus lazertinib(In the phase III MARIPOSA trial (20) the amivantamab plus lazertinib combination demonstrated a 3-year intracranial progression-free survival (PFS) rate of 36% versus 18% with osimertinib monotherapy), and the development of novel EGFR-TKIs with enhanced central nervous system penetration, to further improve outcomes in patients with EGFR-mutant NSCLC complicated by central nervous system metastases.

## Data Availability

The original contributions presented in the study are included in the article/**Supplementary Material**. Further inquiries can be directed to the corresponding author.

## References

[1] London D Patel DN Donahue B Navarro RE Gurewitz J Silverman JS . The incidence and predictors of new brain metastases in patients with nonsmall cell lung cancer following discontinuation of systemic therapy. J Neurosurg. (2022) 137:544–54. doi: 10.3171/2021.9.jns212150 34891140

[2] Thakkar JP Kumthekar P Dixit KS Stupp R Lukas RV . Leptomeningeal metastasis from solid tumors. J Neurol Sci. (2020) 411:116706. doi: 10.1016/j.jns.2020.116706 32007755

[3] Ge Y Xu B Wu H Zhang X Wu J Li J . Efficacy and safety of EGFR tyrosine kinase inhibitors combined with cranial radiotherapy for brain metastases from non-small-cell lung cancer: a protocol for a systematic review and meta-analysis. BioMed Res Int. (2022) 2022:6531748. doi: 10.1155/2022/6531748 35872868 PMC9301690

[4] Ozair A Wilding H Bhanja D Mikolajewicz N Glantz M Grossman SA . Leptomeningeal metastatic disease: new frontiers and future directions. Nat Rev Clin Oncol. (2025) 22:134–54. doi: 10.1038/s41571-024-00970-3 39653782

[5] Remsik J Boire A . The path to leptomeningeal metastasis. Nat Rev Cancer. (2024) 24:448–60. doi: 10.1038/s41568-024-00700-y 38871881 PMC11404355

[6] Le Rhun E Weller M van den Bent M Brandsma D Furtner J Rudà R . Leptomeningeal metastasis from solid tumours: EANO-ESMO clinical practice guideline for diagnosis, treatment and follow-up. ESMO Open. (2023) 8:101624. doi: 10.1016/j.esmoop.2023.101624 37863528 PMC10619142

[7] Wilcox JA Chukwueke UN Ahn MJ Brastianos PK Chukwueke C Dietrich J . Leptomeningeal metastases from solid tumors: A Society for Neuro-Oncology and American Society of Clinical Oncology consensus review on clinical management and future directions. Neuro Oncol. (2024) 26:1781–804. doi: 10.1093/neuonc/noae103 38902944 PMC11449070

[8] Sharma A Low JT Kumthekar P . Advances in the diagnosis and treatment of leptomeningeal disease. Curr Neurol Neurosci Rep. (2022) 22:413–25. doi: 10.1007/s11910-022-01198-3 35588045

[9] Clarke JL Perez HR Jacks LM Panageas KS DeAngelis LM . Leptomeningeal metastases in the MRI era. Neurology. (2010) 74:1449–54. doi: 10.1212/wnl.0b013e3181dc1a69 20439847 PMC2871005

[10] Kermanshahi N Hamidi N Weisberg J Beg U Dabrowski M . The prevalence of seizures in brain metastasis patients on anticonvulsant prophylaxis: A systematic review and meta-analysis. World Neurosurg. (2024) 183:e613–24. doi: 10.1016/j.wneu.2023.12.154 38199459

[11] Kokavec A . A case of MRI-negative leptomeningeal disease from non-small cell lung cancer. Cureus. (2024) 16:e56727. doi: 10.7759/cureus.56727 38646403 PMC11032734

[12] Vogelbaum MA Brown PD Messersmith H Brastianos PK Burri S Cahill D . Treatment for brain metastases: ASCO-SNO-ASTRO guideline. J Clin Oncol Off J Am Soc Clin Oncol. (2022) 40:492–516. doi: 10.1200/gdl.21.1221 34932393

[13] Le Rhun E Guckenberger M Smits M Dummer R Bachelot T Sahm F . EANO-ESMO clinical practice guidelines for diagnosis, treatment and follow-up of patients with brain metastasis from solid tumours. Ann Oncol Off J Eur Soc For Med Oncol / ESMO. (2021) 32:1332–47. doi: 10.1016/j.annonc.2021.07.016 34364998

[14] Hosoda T Katayama M . Epidemiology and prognosis of ommaya reservoir-related bacterial meningitis in adult patients with leptomeningeal metastases from solid tumors: a 10-year retrospective single-center study in Japan. J Infect Chemother. (2021) 27:486–91. doi: 10.1016/j.jiac.2020.10.025 33214071

[15] Tuohy K Dowd R Ali A Badani A Sichinga K Zacharia BE . Medical therapy alone for Ommaya reservoir-associated bacterial meningitis: when it works and when it fails. Neurosurgery. (2025) 97:380–6. doi: 10.1227/neu.0000000000003310 39775083

[16] Pan ZY Song YY Jiang TC Yang X Yang GZ . Clinical trials on intrathecal pemetrexed treated leptomeningeal metastases from solid tumors. Zhonghua Zhong Liu Za Zhi. (2022) 44(1):112–9. 10.3760/cma.j.cn112152-20200711-00647 35073657

[17] Pan Z Yang G He H Zhao G Wang Y Li W . ACTR-65. Intrathecal pemetrexed for patients with recurrent leptomeningeal metastasis from lung adenocarcinoma: a phase I clinical trial (IPRLM, NCT03101579). Neuro-Oncology. (2018) 20:vi26. doi: 10.1093/neuonc/noy148.095

[18] Dossani RH Kalakoti P Thakur JD Nanda A Guthikonda B Bollam P . Ayub Khan Ommaya (1930-2008): Legacy and contributions to neurosurgery. Neurosurgery. (2017) 80:324–30. doi: 10.1093/neuros/nyw031 28173516

[19] Yang Y . (2026). “ Osimertinib with or without chemotherapy as first-line treatment in EGFR-mutant advanced NSCLC with concurrent TP53 mutations (TOP study)”, in: Proceedings of the European Lung Cancer Congress (ELCC) 2026 (Copenhagen, Denmark). Abstract 7MO.

[20] Cho BC Lu S Felip E Spira AI Girard N Lee JS . Amivantamab plus lazertinib in previously untreated EGFR-mutated advanced NSCLC. N Engl J Med. (2024) 391:1486–98. doi: 10.1056/NEJMoa2403614 38924756

